# Supplementary material for: Complexome profiling of the Chlamydomonas psb28 mutant reveals TEF5 as an early PSII assembly factor
Source: Plant Cell. 2025 Mar 17;37(6):koaf055. doi: 10.1093/plcell/koaf055 (PMC12164590; doi:10.1093/plcell/koaf055)
Supplement: koaf055_Supplementary_Data [file koaf055_supplementary_data.zip › psb28_interactive.zip!/plots/intensity/SAR1_Cre11.g468300.t1.2.html]

|  |  |
| --- | --- |
| **Identifier** | Cre11.g468300 |
| **Trivial name** | SAR1 |
| **Euclidean distance** | 3174.50 |
| **Average intensity sum (WT)** | 95.2 || **Average intensity sum (Mut)** | 129.6 |
| **Adjusted euclidean distance** | 24.49 |
| **MapMan annotation** | signalling.G-proteins minor CHO metabolism.others protein.targeting.secretory pathway.unspecified |
| **p-value** (Welch test) of intensity sums | 0.78539 |
